# Increased resistance to sudden noise by audio stimulation during early ontogeny in German shepherd puppies

**DOI:** 10.1371/journal.pone.0196553

**Published:** 2018-05-03

**Authors:** Helena Chaloupková, Ivona Svobodová, Pavel Vápeník, Luděk Bartoš

**Affiliations:** 1 Department of Animal Science and Ethology, Faculty of Agrobiology, Food and Natural Resources, Czech University of Life Sciences Prague, Prague, Czech Republic; 2 Department of Ethology, Institute of Animal Science, Prague, Czech Republic; University of Missouri Columbia, UNITED STATES

## Abstract

The period of early ontogeny constitutes a time when the physical immaturity of an organism is highly susceptible to external stimuli. Thus, early development plays a major role in shaping later adult behavior. The aim of the study was to check whether stimulating puppies at this early stage in life with sound would improve their responsiveness towards unfamiliar noises during the selection process of the police behavioral test for puppies. The cohort comprised 37 puppies from the litters of three mothers. At the commencement of the experiment the dogs were aged 16 days, rising to the age of 32 days at its close. The mothers and litters of the treatment group were either exposed to radio broadcasts, (see below; three litters totaling 19 puppies), while the control group was not exposed to any radio programs (eight litters totaling 18 puppies). All three mothers had previously experienced both auditory circumstances, as described herein. Ordinary radio broadcasts were played to the puppies in the treatment group three times a day for 20 minute periods, always during feeding time. The cohort was subjected to the so-called Puppy Test, i.e. analysis of the potential of each animal, once the dogs had reached the age of 7 weeks. Such tests included exposure to a sudden noise caused by a shovel (100 dB), noise when alone in a room, and response to loud distracting stimuli (the latter two at 70 dB). Said tasks were rated by the same analyst on a scale of 0–5 points; the better the response of the dog, the higher the score given. The differences between the treatment and control groups were analyzed via Mixed Models (PROC MIXED) in SAS. The animals comprising the treatment group responded with a higher score to the sudden noise caused by the shovel than the control dogs (P<0.01). Interestingly, gender was seen to affect response, with the males scoring more than the females (P<0.1). In conclusion, the results suggested that audio stimulation early in life improved the response of the dogs to intense sudden noise, as caused by the shovel. Therefore, acoustic stimulation during the very early period of life has the potential to raise the necessary skills of dogs for military and police purposes, or civilian life.

## Introduction

Early ontogeny is when the physical immaturity of an organism is susceptible and responsive to stimuli. Indeed, adult behavior is profoundly affected during early development by sensory input, motor output, and synthesis of such information [1]. Numerous differences between individuals can be explained by the stimulation methods and enriching experiences encountered early in life [2]. These highlight how poor stimuli (e.g. impaired maternal care, limited social contact, disease, and restricted environment) can negatively impact the young [3–5]. However, pre- and postnatal environments also have the capacity to exert positive influences [5–7]. In previous studies, brief separation or mildly stressful stimulation of neonatal rats caused long-term positive effects. As adults, such rats were less reactive and more emotionally stable than control specimens [8, 9].

In the case of dogs, a puppy is born in a state of extreme neuronal immaturity, after which the nervous system rapidly advances through intense synaptogenesis, wherein external stimulation is fundamental [10]. Within a few weeks, the dog gains a wide range of abilities and motor skills, which over the next few weeks result in some highly crucial developmental pathways in life [11]. In general, the key period for learning starts at 2.5 weeks of age and extends to 12–14 weeks [3]. Puppies are exposed to particular kinds of environmental stimuli, and if these are poor or limited in scope, fearful responses or inappropriate avoidance behavior might be evidenced at a later date [12, 13].

In the 1960s, studies were carried out on early ontogeny in dogs with a view to subjecting puppies to sensory stimulation, wherein remarkable differences were seen in comparison with control (isolated) puppies [3, 14, 15]. Recent evidence shows that tactile or audio and video stimulation during the first week post-partum (pp) decreases fearful reactions towards unfamiliar environments or novel objects [16, 17]. However, the most important impact of handling was found in puppies born and raised in a breeding kennel, where contact with humans was limited, unlike in a family surrounding [16]. Enriching circumstances though simulating handling procedures during early ontogeny has proven useful for preparing military or police dogs, which have to cope with challenging situations [2]. No effect of tactile or thermal stimulation was found in dogs trained for mine detection [18]. One explanation for the unsuccessful findings of said study was the routine daily manipulation conducted with both the control and experimental puppies, which failed to bring about any additional benefit [18]. Despite all this prior research, the authors have not identified anything in the literature that investigates the audio stimulation of puppies and the later response of the same towards sudden noise. For police and military dogs, reactions to sudden noises such as gunshots comprise a fundamental criterion for selecting adult dogs and puppies for future training [19]. Reactions exhibited by dogs that show sensitivity to noise might constitute a serious welfare-related issue, since fear is connected to stressful responses [20], potentially reflected in heightened cortisol concentrations [21]. Herein, the authors tested the hypothesis that the effect of audio stimuli during early ontogeny would improve reactions to noise during the police test for selecting puppies.

## Materials and methods

### Ethics statement

All procedures involving animals adhered to recommendations in the “Guide for the Care and Use of Animals” by the Czech University of Life Sciences Prague. The protocol of the experiment was approved by the Czech Central Committee for the Protection of Animals (Permit number: 63479/2016-MZE-17214). The Police Breeding Facility of the Czech Republic as the owner of the German shepherd dogs gave permission for their animals to be studied.

### Animals and housing

As mentioned above, German shepherd dogs from the Czech Republic Police Breeding Facility (CRPBF), in Prackovice nad Labem, were utilized for study purposes, all of which were owned by the Police Force of the Czech Republic. The cohort comprised 37 puppies from three mothers. After delivery, the mothers and litters in the treatment group were exposed to radio broadcasts (three litters totaling 19 puppies), while the control group did not experience such stimulus (eight litters totaling 18 puppies). The unequal number of the litters was arrived at in order to obtain a comparable number of puppies in both groups. So as to eliminate the potential effect of the three mothers, all of them had experienced both situations in previous seasons. The dogs were kenneled in a parturition room (5 m^2^), and from the age of 4 weeks they had the possibility to access an outdoor pen (225 m^2^).

The puppies were weighed once a week, dewormed at 8–9 days, as well as at 3, 5 and 7 weeks, and weaned off milk at the age of 7 weeks. The puppies were marked with cuts in their fur, and at 7 weeks they were tattooed and micro-chipped. During the first 3 weeks post-partum, contact with humans was limited to receiving treatment, weighing, encouraging feeding by the mothers and the kennels being cleaned after delivery. The puppies were walked outside the kennel without their mothers from the age of 3weeks.

### Treatment

At the age of 16 days, the puppies were exposed to the output of a national radio station, Radiožurnál (a mixture of spoken word and music). The loudspeaker was situated in the open window of the kennel 120cm above the floor, and volume was set to approximately 80 dB. The radio was turned on three times per day for twenty-minute intervals (at 8:00, 13:00 and 18:00), always during supplemental feeding of the puppies. Exposure to the radio ceased when the puppies were aged 32 days. As mentioned previously, the control group did not experience such auditory stimulation.

### Puppy selection test

A test that investigates specific behavior in puppies for selection purposes, the so-called Puppy Test [19] has been routinely applied to assess the potential working ability of police dogs in the Czech Republic for more than 20 years. Herein, the puppies were tested before their feed in the morning at 48.8 days ± 0.8 (mean ± S.E), under circumstances of no precipitation and temperatures in the range of -15°C to +25°C. As described earlier [19], the Puppy Test is based on 10 short tasks: independent movement and interaction with the analyst; negotiating obstacles; response to distracting auditory stimuli caused by a shovel (100 dB); entering a room; behavior toward a person; behavior in new environments; response to a distracting noise while left alone in a room (70 dB); response to loud distracting stimuli (70 dB); retrieval; and a tug-of-war (for a detailed description of all tasks, see S1 Table). Each task was rated by the same analyst (PV), who awarded points on a 0–5 scoring system; the better the response, the higher the score. All the puppies were tested separately from other conspecifics.

The loudness of the auditory tasks, i.e. distracting auditory stimuli caused by a shovel, the distracting noise while left alone in a room, and loud distracting stimuli were measured on a digital audiometer, model SL-400 by Voltcraft^®^. In accordance with the advance hypothesis of the authors, attention was focused on elements involving measurement of response to sudden noise.

## Statistical analyses

All data were analyzed using SAS (version 9.4). The results were considered statistically significant when P ≤ 0.05. Differences between the control and treatment groups in response were gauged by applying the Generalized Linear Mixed Model (GLMM, PROC MIXED in SAS). The assumption made was that repeated measures pertaining to a subject would correlate (SUBJECT = ID of a female in the REPEATED statement), and repeated measures between subjects would be independent (Treatment in the REPEATED statement). In accordance with Littell and Pendergast [22], candidate covariance models were compared with various covariance structures. Based on the Akaike information criterion AIC [23] and Schwarz’s Bayesian information criterion BIC [24], the CS covariance model was the best fit for the GLMM. The significance of each fixed effect in the GLMM model was assessed by carrying out an F-test. All analyses were performed such that an individual puppy represented a statistical unit. The points from the individual tasks in the Puppy Test were log-transformed to improve normal distribution (checked for normality by the Kolmogorov-Smirnov test).

Scores were entered into the GLMM as dependent variables, derived from the following auditory tasks: response to distracting stimuli caused by a shovel (sudden noise by a shovel); response to distracting sounds while left alone in a room (noise when alone); and response to loud distracting stimuli (loud noise in a room). Fixed effects were represented by classes of treatment (i.e. the treatment or control group), and sex (male or female), while continuous variables comprised weight of the puppy during the test (0.05 to 0.15 kg), age (48 to 50 days), and interactions between the aforementioned fixed effects.

Least squares means were calculated by computing the mean for each class effect and averaging the means of such class effects. Said averages of such means were then applied to compare effects. Hence, the means were adjusted for the number of observations of each treatment. This estimate was unbiased since it took into account the unequal number of observations. Least-squares means (referred to hereafter as adjusted means) were computed for each class, and variations between the classes were gauged by conducting a t-test. The authors applied Tukey adjustment for multiple comparisons.

## Results

**Fig 1:**
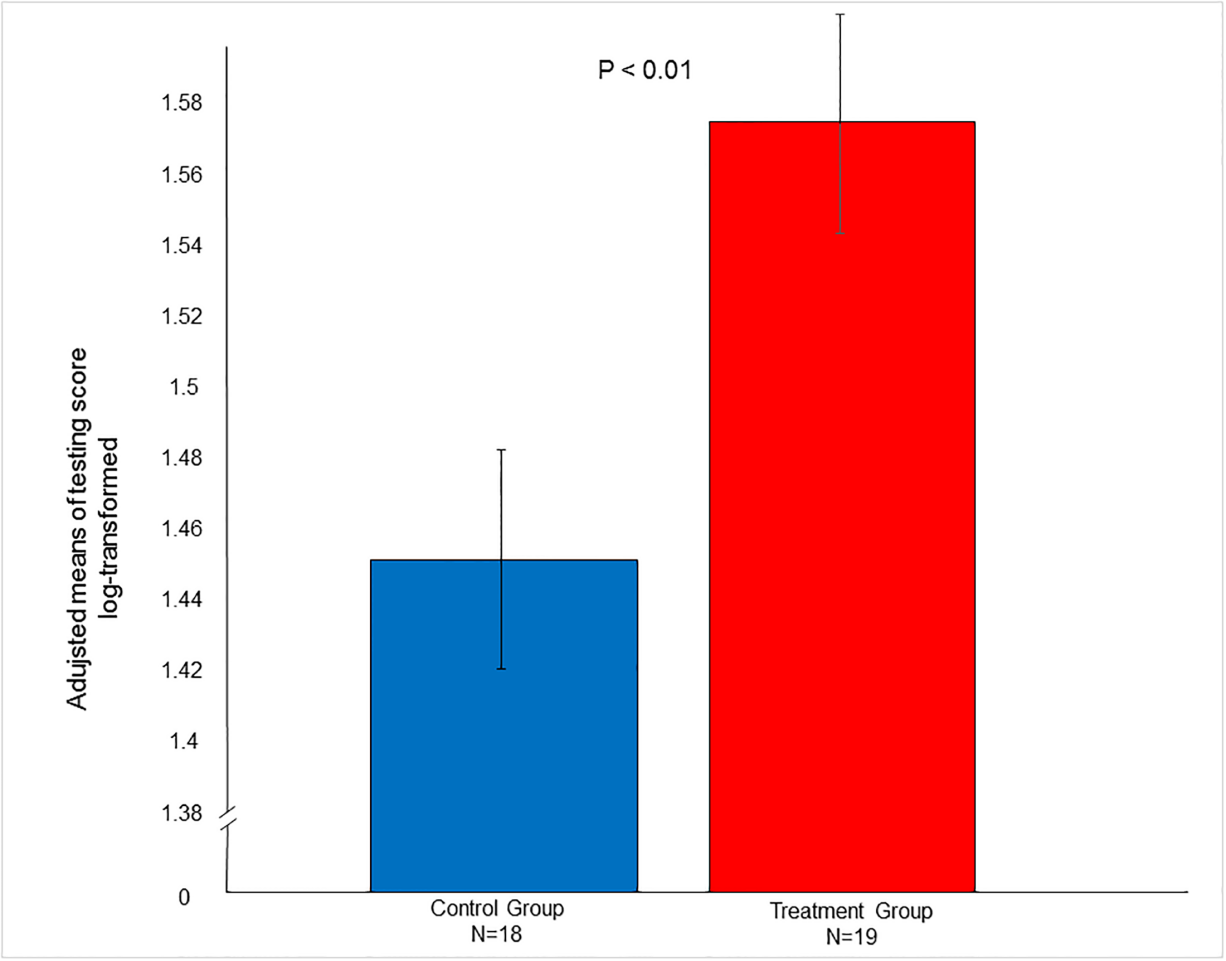
Effect of treatment pertaining to response to sudden noise caused by a shovel.

**Fig 2:**
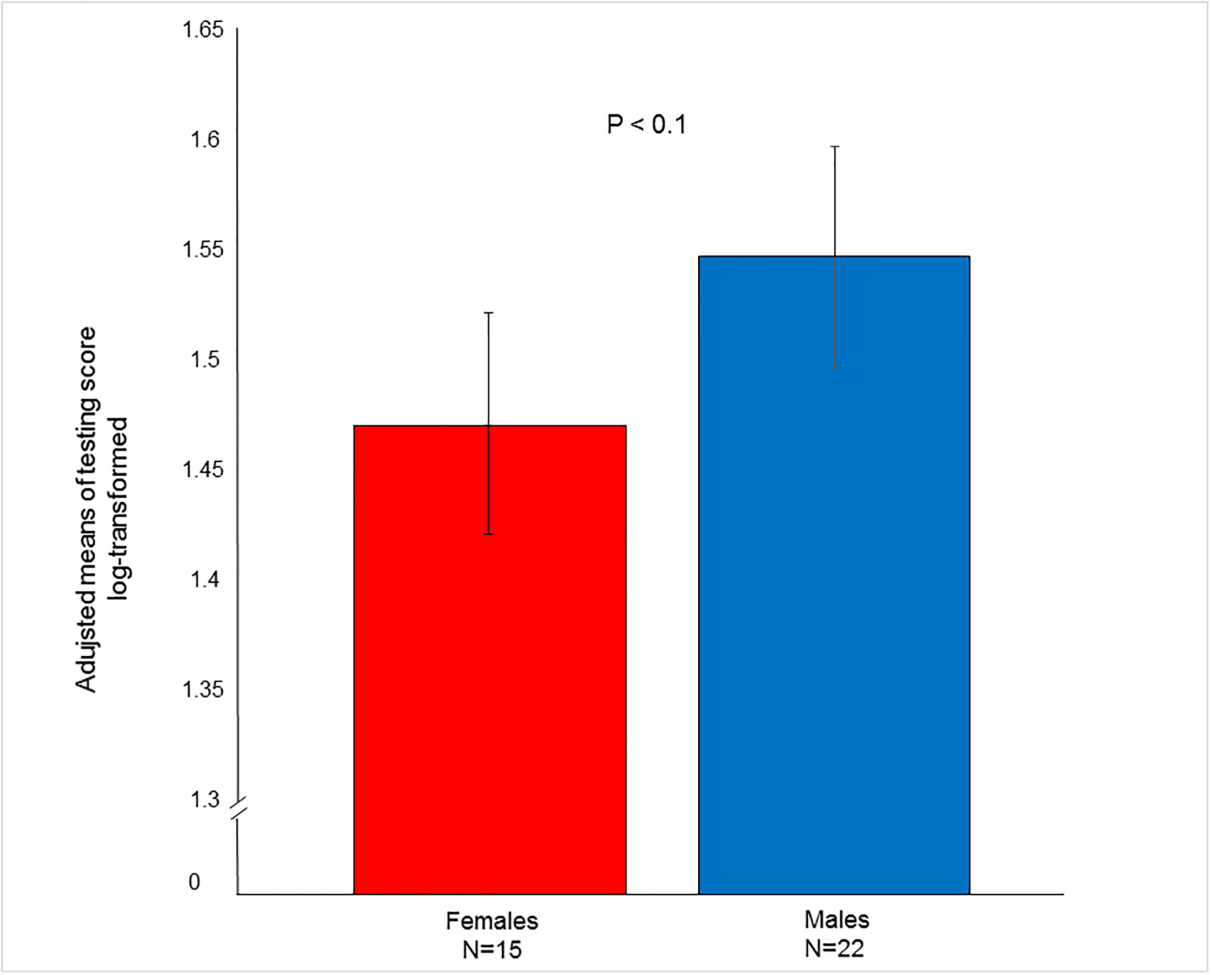
Effect of sex of the puppies pertaining to response to sudden noise caused by a shovel.

The three noise-related tasks (i.e. sudden noise by a shovel, noise when alone and loud noise in a room) were inter-correlated (rs = 0.44 to 0.52), after which the authors decided to concentrate solely on the effect of treatment by sudden noise by a shovel. Indeed, the response to the same was seen to be significant (F_1,33.2_ = 7.98; P < 0.01). The treatment group demonstrated a higher score in said task than the control group (Fig 1). The influence of gender on the score was discerned (F_1,32.8_ = 2.98; P = 0.09), the males achieving a higher score than the females (Fig 2).

## Discussion

The results derived from the experiment suggest that audio stimulation can affect the reaction of a puppy in early life said sudden noise caused by a shovel, which characterized the most intense sound (100 dB) in the puppy selection test [19], as tested when the puppies were at 7 weeks of age.

In general, studies in the literature have described clearly positive stimulation is achieved in puppies handled to a great degree versus socially deprived groups, thereby reducing anxiety and enhancing problem-solving skills in the former of the two (e.g. [12]); this is also seen to a moderate level in groups undergoing audio-visual stimulation versus a control group [17]; as well as if animals are gently handled on a daily basis for periods of 5 minutes [16], wherein the most significant results were found for puppies raised in a breeding kennel at which contact with humans was limited versus puppies cared for by families at home [16]. Nevertheless, an experiment on the early neurological stimulation of puppies (aged 3–16 days), which were to undergo training for detecting mines, failed to show any real variation between the groups [18]. Therein, aspects included tactile stimulation, head manipulation, supine position, and thermal stimulation (by methods described in [2]). The insignificant results of the latter study were considered to stem from such exercises not ultimately providing additional benefit to puppies already experiencing socially and stimulatory rich environments [18]. Returning to the present paper, despite the fact that the puppies were handled on a daily basis, it was still possible to discern a benefit through the acoustic stimulation described. This was demonstrated in the responses given by the animals in the treatment group towards a sudden noise compared to those in the control group that had not received such stimuli. It is possible that the insignificant results of the study by Schoon and Berntsen [18] could be due to exposure to the challenge manipulation outlined therein [18] prior to commencing the socialization period [1, 25], the former occurring when the sensory development of the puppies was still very low [1]. In contrast, the results reported in the current study, just like in research by Pluijmakers and Appleby [17] and Gazzano and Mariti [16], show that auditory treatment of puppies has an effect in approximately the 4^th^ week of the life of a puppy, when its senses are quite well developed [1].

In terms of gender, the male puppies of the cohort tended to exhibit a lesser response to the sudden noise caused by a shovel, thereby gaining a higher score than the females. At the age of 8 weeks, Wilsson and Sundgren [26] reported that their female puppies exhibited a longer latency for yelping, were more active, or visited more objects in the arena utilized than the males. This suggested that the females responded better than the males to stressful stimuli. A possible explanation for this could be the more rapid maturation of females over males of the same age [27]. However, gender was not discerned to play a role in approximately 300 puppies undergoing an open field test at the age of 8 weeks [28]. Nevertheless, responsiveness towards noise was not investigated in any of these three studies [26–28]. Therefore, the question remains if the potentially stronger response to sudden noise indicated for female puppies is attributable to differences in ontogenetic development.

## Conclusion

The present study shows that puppies experiencing short-term exposure to radio broadcasts can benefit from an enhanced future response to a sudden noise. Thus, acoustic stimulation during such an early period of life has the potential to elevate the necessary skills of dogs intended for the military or police force, and maybe even for civilian applications.

## Supporting information

S1 Table The puppy selection test. Description of tasks and evaluation of behavior.
(DOCX)

S1 Data Supporting information data set. (XLSX)
