# Supplementary material for: Increased resistance to sudden noise by audio stimulation during early ontogeny in German shepherd puppies
Source: PLoS One. 2018 May 3;13(5):e0196553. doi: 10.1371/journal.pone.0196553 (PMC5933723; doi:10.1371/journal.pone.0196553)
Supplement: S1 Table — Description of tasks and evaluation of behavior. (DOCX) [file pone.0196553.s001.docx]

| Tasks | Description |
| --- | --- |
| 1. Independent movement and interactions with the tester | The tester evaluated responses of the puppy to distracting stimuli (such as hand claps) while being led along the testing route of 50 m. The closer to the tester the puppy was and the more interactions with the tester it initiated, the higher the score |
| 2. Negotiating obstacles | The tester evaluated the animal’s speed and hesitation in overcoming the 20 cm wooden obstacle and climbing up and down 5 concrete steps. The higher the speed with less hesitation, the higher the score |
| 3. Response to distracting auditory caused by a shovel | Just before entering a room, at a distance of approximately 1 m from the puppy, the tester initiated a loud noise by hitting a metal sewer lid with a shovel three times in a series of three hits (total of nine), within 30 seconds. Running away and showing fear was scored 0. With less pronounced fear and increased exploratory interest in the noise, the higher the score. |
| 4. Entering a room | The courage of the puppy when entering an unknown room (10 m x 1.4 m x 2.5 m with 3 windows 1 m x 1 m) and its behavior, while being left alone there, were scored. The less time it took the puppy to enter the room, the higher the score. |
| 5. Behavior toward a person | Behavior of the puppy toward a familiar tester who entered the room and remained motionless was evaluated. If the puppy did not display any distinct reaction, it was provoked by the tester using movement, calling, or petting. Behavior of the pup toward the tester in other tests was also scored as a part of this test. The more the puppy approached the tester in a friendly manner, the higher the score. |
| 6. Behavior in new environments | The exploring activity of the puppy left alone by the tester in a closed room (the same as above) for 2 min was scored. Any exploration for the rest of the testing period (another 8 min) was additionally assessed. The more movement and exploratory behavior, the higher the score. |
| 7. Response to a distracting noise while left alone in a room | The reaction of the pup to noises caused by repeated knocking on the window, while left alone by the tester in a closed room, was evaluated. Running away and showing fear was scored 0. With less pronounced fear and increased exploratory interest in the noise, the higher the score. |
| 8. Response to loud distracting stimuli | After returning of the tester to the room with the puppy, the tester initiated loud, sudden noises by hitting a weight twice against a table top. Reactions of the puppy to loud noises in the presence of a person were evaluated. Running away and showing fear was scored 0.With less pronounced fear and increased exploratory interest in the noise, the higher the score |
| 9. Retrieval | A tennis ball was tossed away from each puppy. Willingness to chase, catch, and fetch the ball were scored. The more chasing, catching, and fetching of the ball, the higher the score. |
| 10. Tug of war | The test was aimed at the courage and hunting behavior of the puppies while exposed to a rag being drawn away from them. Ability of the pup to grip and fight with the rag being drawn away from it was scored. The higher vigor in the response, the higher the score. |
